# Poly(lactic acid) Degradation by Recombinant Cutinases from *Aspergillus nidulans*

**DOI:** 10.3390/polym16141994

**Published:** 2024-07-12

**Authors:** Eric Alvarado, Rafael Castro, José Augusto Castro-Rodríguez, Arturo Navarro, Amelia Farrés

**Affiliations:** Departamento de Alimentos y Biotecnología, Facultad de Química, UNAM, Mexico City 04510, Mexico; roccoeam@comunidad.unam.mx (E.A.); castros.rafa98@comunidad.unam.mx (R.C.); jaugusto_09roca@comunidad.unam.mx (J.A.C.-R.); arturono@unam.mx (A.N.)

**Keywords:** poly(lactic acid) degradation, cutinase, *Aspergillus nidulans*, lactic acid

## Abstract

Poly(lactic-acid) (PLA) is a biodegradable polymer widely used as a packaging material. Its monomer, lactic acid, and its derivatives have been used in the food, cosmetic, and chemical industries. The accumulation of PLA residues leads to the development of green degrading methodologies, such as enzymatic degradation. This work evaluates the potential use of three cutinolytic enzymes codified in the *Aspergillus nidulans* genome to achieve this goal. The results are compared with those obtained with proteinase K from *Tritirachium album*, which has been reported as a PLA-hydrolyzing enzyme. The results show that all three cutinases act on the polymer, but ANCUT 1 releases the highest amount of lactic acid (25.86 mM). Different reaction conditions assayed later led to double the released lactic acid. A decrease in weight (45.96%) was also observed. The enzyme showed activity both on poly L lactic acid and on poly D lactic acid. Therefore, this cutinase offers the potential to rapidly degrade these package residues, and preliminary data show that this is feasible.

## 1. Introduction

Polymers have been used by humans for a very long time. Initially, natural products, like rubber, were used. In the mid-twentieth century, advances in chemistry led to the synthesis and diversification of synthetic polymers. These have played a prominent role in everyday life and in different sectors, such as the food, chemical, medicine, construction, and locomotion industries. They were designed to have low prices and a long functional life and have caused a serious environmental problem due to residue accumulation because of their recalcitrant nature [1,2,3].

Among the most used plastics are polyesters. They can degrade naturally faster than other plastics, as the ester bond is attacked by several naturally occurring enzymes. Their monomers can be chemically synthetized from oil derivatives or by biotechnological means. Poly(lactic acid) (PLA) is considered a biodegradable polyester as it degrades in a shorter time span. Therefore, demand for it has grown, especially in food and beverage packing materials [4]. It is an aliphatic homopolymer formed by lactic acid monomers [5]. It can degrade naturally, but microorganisms and enzymes were found to degrade it more than two decades ago, when Ikura [6] isolated microorganisms able to grow on it, and then Nakamura [7] reported that a cutinase from Amycolitopsis degraded PLA. Some years later, another cutinase from Cryptococcus was reported to perform this reaction. Initially, it was thought that it was a lipase [8]. The similarities to the catalytic site and mechanism in other esterases have resulted in candidates for PLA degradation among serine proteases, lipases, esterases, and cutinases [9,10,11]. Proteinase K, produced by *T. album*, was reported to degrade PLA and release lactic acid, and the list of microorganisms and enzymes so far reported has grown. It includes enzymes like lipase, produced by *Bacillus safaensis* isolated from landfills [12], or cocultures, which could be useful to degrade multiplastic composites; an example contains *Pseudomonas mendocina* and *Actinomucor elegans* [13]. Furthermore, some new developments, such as genes isolated from metagenomes [14] or assays with bacterial consortia, offer new opportunities. For example, the use of a bacterial consortium combined with the use of deep eutectic solvents (DESs) has been shown to degrade both PLA and poly (ethylene terephthalate) (PET) [15] in a landfill. However, the development of circular economy processes is attractive, as monomers would be recovered and new, high-value products could be produced [16]. To achieve this, efficient enzymes must be isolated or engineered. In search of better catalysts, this work explores the possible degradation of this polymer by a member of the genus Aspergillus, a genus that has been reported to degrade PLA [17]. This saprophytic mold encodes more than sixty esterases in its genome and four cutinases. Their different biological roles in the action over cutin has been described elsewhere [18], forming a cutin-degrading system that includes a constitutive enzyme (ANCUT3), two inducible enzymes (ANCUT1 and ANCUT2), and a stress-responding enzyme (ANCUT4). They are phylogenetically related to other fungal cutinases, and the highest-identity in amino-acid sequence between these cutinases is that found between ANCUT1 and ANCUT 2 [19]. Due to the low level of production in the native system, and the possibilities that cloned and engineered enzymes offer in developing better catalysts, these enzymes have been cloned in *Pichia pastoris* (*Komagatella phaffi*) [20] and can be produced in sufficient amounts in instrumented reactors. The results presented in this work indicate that ANCUT1 yields the best results, leading to higher lactic acid production and weight loss, even higher than those obtained by Proteinase K, which is available commercially as a molecular biology reagent.

## 2. Materials and Methods

### 2.1. Recombinant Enzyme Production

The genes encoding cutinases ANCUT1, ANCUT2, and ANCUT3 from *A. nidulans* PW1 were cloned in the plasmid pPICZα in *Pichia pastoris X33,* as described previously [19]. The recombinant clones were grown in culture media BMGY (buffered glycerol complex medium), BMMY (buffered methanol-complex medium), YPD (yeast extract, peptone, and dextrose), and YP (yeast extract and peptone), prepared as described [21]. The strains were stored in glycerol at −70 °C, and 200 µL from this stock was inoculated into sterile 25 mL YPD/zeocin (0.01%) in a 250 mL Erlenmeyer flask and incubated for 24 h at 29 °C and 300 rpm in an orbital shaker (Innova 40, New Brunswick Scientific, Edison, NJ, USA). The culture was centrifuged for 10 min at 7500 rpm in a centrifuge (Biofuge, Heraeus, Thermofisher Scientific, Santa Clara, CA, USA), and the cell pellet was resuspended in 2 mL YP medium to achieve a DO_600 nm_ of 1 when inoculated into 25 mL of sterile BMGY medium in a 250 mL Erlenmeyer flask. It was incubated for 24 h at 29 °C, as previously described. The culture was centrifuged and washed twice with YP medium, the supernatant was discarded, and the cell pellet was resuspended in 2 mL YP medium and added to sterile BMMY medium. It was incubated as described previously, and 1% methanol was added after 24 h to induce enzyme production. Incubation proceeded for another 24 h, and then the culture was centrifuged, and the supernatant was used to assay protein via the Bradford assay, as well as enzymatic activity, as described below. The supernatants were concentrated by ultrafiltration with a 10 kDa membrane.

**Proteinase K preparation.** According to the provider’s instructions, 15 mg lyophilized proteinase K (GIBCOBRL) was dissolved in 5 mL of Tris-HCl 50 mM buffer, pH 8, and 20 mM CaCl_2_ was added. Soluble protein and carboxylesterase activity were assayed as described below.

**Microplate protein assay.** Protein was assayed according to Bradford [22] in microplates. Samples were diluted so that absorbance readings fell in the valid range. In each well, 160 µL of diluted sample was placed, with 40 µL Bradford reagent (Bio Rad, Hercules, CA, USA), and a blank containing 160 µL distilled water and 40 µL Bradford reagent were used. The mixtures were homogenized for 5 min at room temperature. The samples were analyzed in an Epoch BioTeK (Winooski, VT, USA) spectrophotometer, absorbance was read at 595 nm, and the results were processed with the software Gen 5 1.10 and compared to a bovine serum albumin (BSA) standard curve with a concentration range between 1.6 μg/mL and 10.2 μg/mL.

**Carboxylesterase microplate assay.***p*-nitrophenylbutyrate (*p*-NFB) was used as substrate. In each microplate well, 170 µL phosphate buffer 50 mM, pH 7, 10 µL of the BMMY culture supernatant, and 20 μL of 1 mM *p*-NFB were placed. A blank using phosphate buffer instead of culture supernatant was used to evaluate autohydrolysis. Triplicates were run for all samples, reading absorbance at 420 nm. Reaction kinetics was followed for 10 min, and readings were taken every minute, as indicated by software Gen 5 1.10, in an Epoch BioTek spectrophotometer. One activity unit (U) is defined as enzyme that converts 1 µmol *p*-NFB to p-nitrophenol (p-NP) per minute under the assay conditions. A standard p-nitrophenol curve was built to perform the assay.

**Protein profile (SDS-PAGE)** (sodium dodecyl sulfate–polyacrylamide gel electrophoresis). Denaturing polyacrylamide gels were prepared, using sodium dodecyl sulphate (SDS) as detergent and *β*-mercaptoethanol as a reducing agent, according to Laemmli [23]. A 4% *bis*-acrylamide stacking gel was prepared, and a 12% separation gel was used. Supernatant samples were treated with *β*-mercaptoethanol and loaded into the gel. Molecular mass marker was used as reference (180–10 kDa, Thermo Fisher Scientific, CA, USA), and MiniProtean II or IV (Bio Rad, Hercules, CA, USA) electrophoresis equipment was used at 80 Volts for 20 min and, afterwards, at 180 Volts, at 4 °C, with running buffer. Gels were stained with Coomassie blue.

**Zymogram.** Esterase activity was observed in polyacrylamide gels with no *β*-mercaptoethanol and without heating the samples, under the conditions described above. Zymograms were prepared according to Karpushova [24]. After the gel electrophoresis was completed, the gels were incubated in a 50 mM phosphate buffer, pH 7, and 0.5% Triton X-100 for 30 min, to allow enzyme renaturation. The solution was changed, and a solution containing α-naphthyl acetate was used for 30 min, with agitation at room temperature. The gel was washed with distilled water and submerged in a Fast Red-containing solution until brown bands were observed, which indicate carboxylesterase enzyme activity. Finally, the gel was washed with distilled water.

### 2.2. Poly L-Lactic Acid (PLLA) Hydrolysis Reaction with Recombinant Cutinases

The enzymatic hydrolysis reaction was performed by placing the enzyme in a 2 mL vial with 10 mg powder PLLA passed through a #20 sieve (850 μm), together with the necessary volume of the concentrated enzyme supernatant that had 100 µg of protein (1% *w*/*w* enzyme/polymer). A 1 mL final volume was achieved with the respective buffer. Blanks contained buffer and PLLA with no enzyme to assess the buffer’s role in degradation, as well as another with enzyme without PLLA to assess the effect of the enzyme concentrate. The vials were incubated according to the following hydrolysis conditions: 37 °C, sodium phosphate buffer 50 mM, and pH 7 for ANCUT 1 and ANCUT2, while Tris-HCl buffer 50 mM, pH 9, was used for ANCUT3. Degradation was assayed after seven days by measuring the amount of lactic acid released to the reaction medium, as described below.

### 2.3. PLLA Hydrolysis with Proteinase K

The reaction mixture was prepared as described above. Tris-HCl 50 mM buffer, pH 8, was used, adding CaCl_2_ 20 mM, and the vials were incubated at 37 °C. Degradation was assayed after seven days by measuring the amount of lactic acid released to the reaction medium, as described below.

### 2.4. Assay of Lactic Acid after Enzyme Hydrolysis of PLLA

The L-lactate Assay kit (Sigma Aldrich MAK-329, St. Louis, MO, USA) was used. A standard curve with dilutions of a 2 mL lactic acid standard were prepared according to the provider instructions. A reaction mixture was prepared with the rest of the reagents provided, and it consisted of 60 µL of this reaction mixture added to a microwell containing 20 µL of each dilution. Initial absorbance was determined at 565 nm, the sample was incubated for 20 min at room temperature, and the final absorbance was read on an Epoch spectrophotometer (Biotek, Winooski, VT, USA). The standard curve was built, plotting the differences between final and initial absorbance vs. L-lactate concentration. All the samples were processed in the same way

### 2.5. Assay of Lactic Acid after Enzyme Hydrolysis of PDLA

An amount of 10 mg of PDLA (poly D L lactic acid) (15,000 g/mL, Sigma Aldrich) was incubated at 40 °C, in 648 µL of 50 mM Tris HCl buffer, pH 9, and 342 µL of enzyme extract, containing 10,000 U ANCUT1. After 7 days, reaction mixtures were diluted 1:25, and 20 µL were placed in a microwell of a 96-microwell plate with 80 µL of the respective enzymatic reaction kit to assay colorimetrically; absorbance was measured at 565 nm on an Epoch spectrophotometer (Biotek, Winooski, VT, USA) with either the L or D isomer (L-Lactate Assay kit, Sigma Aldrich MAK-329) or D-Lactate Assay kit (Sigma Aldrich (MAK-336)). A reading was taken initially, and a final one was taken after 20 min of reaction.

### 2.6. Weight Loss

Polymer films were washed with ethanol after the enzymatic reaction occurred, and they were dried in an oven at 37 °C overnight. Their weight was assessed using an analytical balance, and weight loss was calculated as follows:

(1) $$Weight\;loss\;\left(\%\right)=\frac{(Weight\;of\;material\;not\;subject\;to\;degradation-Weight\;of\;material\;subject\;to\;degradation}{Weight\;of\;materiall\;not\;subject\;to\;degradation}\times 100$$

### 2.7. Morphological Changes Detected by Scanning Electronic Microscopy (SEM)

The polymer films, whether treated with enzymes or untreated, were washed with ethanol and dried in an oven at 37 °C for one week. The film morphology was examined using SEM (JSM-5900LV) (JEOL, Pleasonton, CA, USA), at high vacuum, with an acceleration voltage of 20 kV. The films were covered with a thin gold film (10 Å) before observation.

**Statistical analysis.** The samples were analyzed with one-way analysis of variance (ANOVA) and Tukey’s test to determine the significant differences among treatments. Differences were considered significant if the *p* value was lower than 0.05. Minitab software was used (Minitab 20.3 (64-bit) software, Minitab® 2021).

## 3. Results

### 3.1. Enzyme Production and Activity Assay

**Figure 1:**
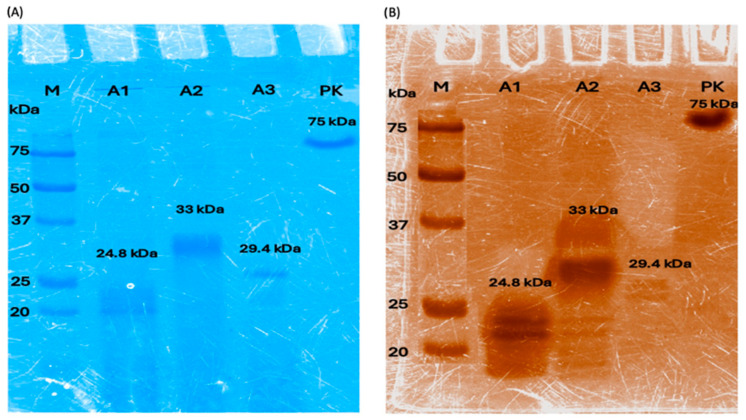
(**A**) Protein profile of the crude supernatants obtained from *P. pastoris* and the commercial preparation of Proteinase K. SDS-PAGE stained with Coomassie blue. Lane M, molecular mass marker; lane A1, ANCUT1; lane A2, ANCUT 2; lane A3, ANCUT3; lane PK, Proteinase K. (**B**) Zymograms of the same samples. Esterase activity is shown using α-Naphtyl acetate as substrate. The expected molecular masses are shown in each lane.

The clones containing each of the ANCUT genes were cultured as described above. The production of the enzymes of interest was monitored by performing a protein profile of the crude culture supernatant in SDS-PAGE gels, including a molecular weight marker. A zymogram was also performed to verify enzyme activity “in situ” (Figure 1A,B). As can be seen, the molecular masses detected for each of the recombinant enzymes correspond to those detected previously in the work group (ANCUT1, 24.8 kDa; ANCUT 2, 33 kDa; and ANCUT3, 29.4 kDa) [25,26] and to their predicted molecular masses according to the gene sequences. The same is true for proteinase K (75 kDa).

We evaluated esterase activity using p-NPB as a substrate. The activities obtained were different for each enzyme: ANCUT2 showed the highest activity, as shown in Table 1. However, the protein concentration was similar for each cutinase.

### 3.2. PLLA Degradation Assayed through L-Lactic Acid Release

**Figure 2:**
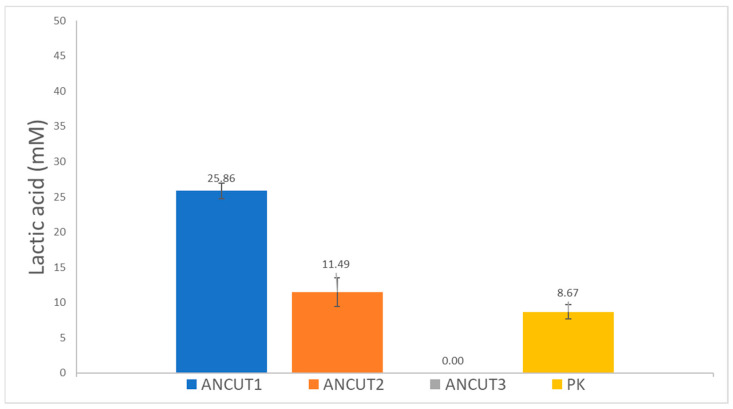
Released lactic acid after PLLA hydrolysis with different recombinant cutinases and proteinase K. The results were obtained after 7 days of incubation with 10 mg powder PLLA passed through sieve # 20 (particle size, 850 µm), 1% enzyme, the buffer indicated for each enzyme, no agitation, and 0.005% sodium azide.

After incubation for 7 days with powder PLLA that was recovered after passing through a #20 sieve (850 μm), and with the enzymes, the released L-lactic acid was assayed. The results in Figure 2 show that ANCUT1 released the highest amount of L-lactic acid, 25.86 mM, followed by ANCUT2, which released 11.49 mM. However, ANCUT3 did not release any detectable lactic acid, even if some small fibers appear in the reaction medium. These might be oligomers or other products, such as alkyl lactates. Further analysis should be performed to characterize them and to identify the hydrolysis mechanism that ANCUT3 carries out. Table 2 shows that weight loss is achieved with this enzyme. The results obtained for Proteinase K in this work, which is a positive control, correspond to those reported elsewhere [27], but the value of 8.67 mM obtained is lower than those obtained with ANCUT1 and ANCUT 2. The different values obtained for each enzyme are statistically significant according to the tests performed.

### 3.3. Establishment of Conditions for the Enzymatic Degradation Reaction

**Figure 3:**
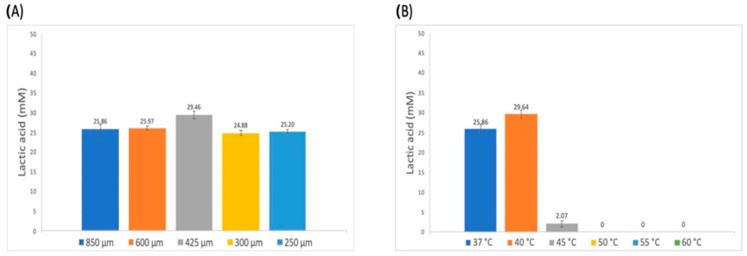

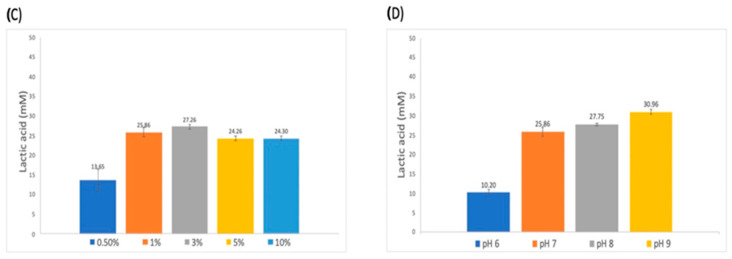
Influence of reaction conditions on PLLA hydrolysis using ANCUT1. (**A**) Effect of particle size on L-lactic acid released. (**B**) Effect of temperature on L-lactic acid released. (**C**) Effect of ANCUT1 concentration on L-lactic acid released. (**D**) Effect of pH on L-lactic acid released using 50 mM sodium phosphate buffer. Bars represent the averages for the data from at least three independent experiments.

Several factors that might influence the degradation efficiency were assayed. The results are presented in Figure 3. The first parameter considered was the mechanical pretreatment of the polymer, by grinding it and passing it through different sieves, which results in different particle sizes. Then, the reaction temperature, as well as the pH and reaction buffer, were evaluated. Finally, the amount of enzyme was considered. All the reactions were incubated statically.

**Figure 4:**
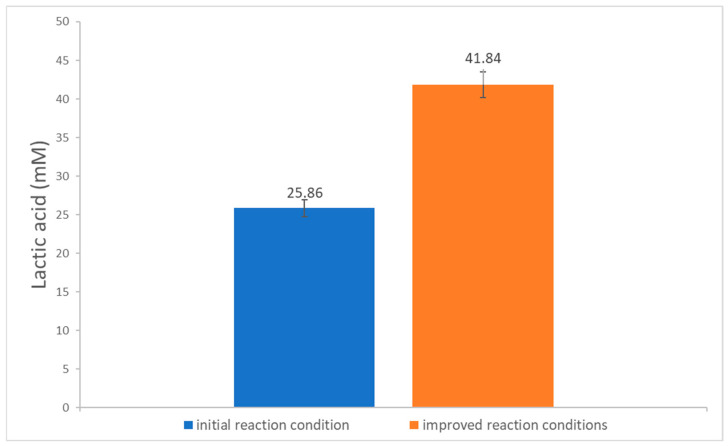
Released lactic acid after PLLA hydrolysis with initial conditions and improved reaction conditions for ANCUT1 3% at 40 °C, 50 mM sodium phosphate buffer, pH 9, 10 mg powder PLLA with 425 μm particle size, and 0.005% sodium azide. The results were obtained after 7 days of incubation.

The best condition for each parameter was selected, and each was included in an integrated experiment using ANCUT1. They were as follows: enzyme concentration, 3% (g_enzyme_ g_PLLA_^−1^); PLLA, 425 μm particle size; 50 mM sodium phosphate; pH 9; 40 °C. As shown in Figure 4, the improved reaction conditions released 41.84 mM lactic acid, which represents a 62% increase compared to the initial experiments and a 483% increase compared to the lactic acid produced by PK.

### 3.4. PLLA Degradation Determined by Weight Loss

A series of reactions was performed in higher volumes to allow for a weight loss assay. The results shown in Table 2 indicate that ANCUT2 and ANCUT3 do not release as much lactic acid as ANCUT1, but they degrade HMW PLLA, causing weight loss (7.43% and 6.93%, respectively). This enzyme yields the highest values of L-lactic acid, as well as the highest weight loss (45.96%). This value for the rate of weight loss falls within the range of other PLA-degrading enzymes [12].

In this experimental series, a commercial packing material based on poly(lactic acid) was included and treated under the same conditions. In fact, a weight loss of 49.7% was achieved, but a very low amount of L-lactic acid (1.94 mM) was detected. Therefore, the detailed composition of the package should be studied, as it might have other components that hinder lactic acid release or contain other materials subject to degradation by ANCUT1.

In an independent experiment, PDLA was subject to the action of ANCUT1, and the results indicate that the enzyme released both L lactic acid and D lactic acid. In total, 16.32 mM L-lactic acid was obtained, while 22.70 mM D-lactic was released, which is 39% higher than the L isomer, which apparently shows that the ANCUT1 enzyme is not enantioselective and acts on both isomers. As can be seen in Table 2, PDLA suffered a considerable weight loss, slightly smaller than that achieved with PLLA. However, more research is needed on the composition of commercial packing materials and the action mechanisms of the enzyme.

### 3.5. Morphological Changes Evaluated by Scanning Electronic Microscopy (SEM)

**Figure 5:**
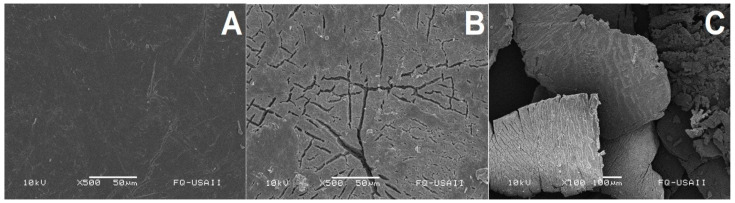
Non-treated PLA (×500) (**A**). PLA treated with ANCUT1 with its best reaction condition after 7 days (×500) (**B**). PLA treated with ANCUT1 with its best reaction condition after 7 days (×100) (**C**).

The PLLA powders degraded by the different enzymes, as well as the commercial package degraded with ANCUT1, were analyzed with SEM. As can be seen in the series of images, non-treated PLLA showed a homogeneous surface, which was smooth, without imperfections (Figure 5A), while those treated with ANCUT1 showed cracking (Figure 5B,C).

**Figure 6:**
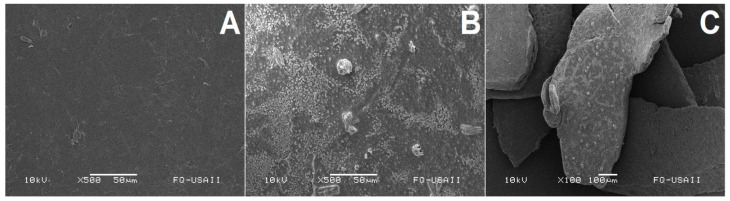
Surface morphology of non-treated PLLA (×500) (**A**). PLA treated with ANCUT2 after 7 days (×500) (**B**). PLA treated with ANCUT2 after 7 days (×100) (**C**).

**Figure 7:**
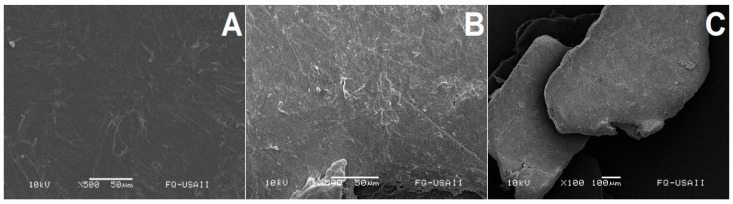
Surface morphology of non-treated PLLA (×500) (**A**). PLA treated with ANCUT3 after 7 days (×500) (**B**). PLA treated with ANCUT3 after 7 days (×100) (**C**).

However, when PLLA was treated with ANCUT 2 or ANCUT 3 (Figure 6 and Figure 7), no clear differences can be observed between the treated and non-treated samples, except for some small points on the surface of the sample treated with ANCUT2.

Both in this case and in that of the PLLA treated with ANCUT 3, no physical changes are detected, even if there is a moderate weight loss

## 4. Discussion

The purpose of this work was to determine whether the cutinases derived from *Aspergillus nidulans* and expressed in *Pichia pastoris (Komagatella phaffi)* were suitable for use in developing a circular economy process to recover lactic acid and, eventually, synthesize higher-added-value products. The results indicate that only one of them, ANCUT1, yields an interesting amount of L-Lactic acid, with values that are higher than those obtained with Proteinase K, which has been reported as a promising PLA-degrading enzyme [11,27]. It must be stated that ANCUT1 yields 0.91 mmol lactic acid/g enzyme/h^−1^, while that reported in the Carbios patent yields 2.23× that amount [28]. The highest yield found in the literature is 100x that obtained with ANCUT1 and corresponds to an enzyme produced by *Pseudozyma anctartica* [29]. Therefore, further work regarding either reaction conditions or the improvement of the characteristics of the enzyme needs to be performed.

An initial effort was made to improve the results, considering the particle size, reaction temperature, pH, and enzyme levels. The particle size of 425 µm yielded the best result in terms of lactic acid release, and this result is significantly different from the other values. The effect of particle size is related to the area of contact between the enzyme and the substrate. The fact that the best value was found for the intermediate size might indicate that there is a compromise between the area reached by the enzyme and diffusion issues for the release of the product. This behavior might be related to that observed with enzyme concentration, as the intermediate level proved to be the level that yielded the highest results. High levels of enzyme might interfere with the mobility required to perform the degradation reaction.

Temperature proved to be a critical factor. The result is different to the optimal temperature that the enzyme shows when assayed as an esterase, which is 50 °C, but its behavior towards pH corresponds to the fact that it is an alkaline cutinase [26].

We do not have enough evidence to determine whether the activity loss at 45–60 °C could be attributable to enzyme instability or to changes in PLLA substrate due to exposure to the temperatures evaluated. The mechanisms and factors that result in enzyme inactivation must be explored systematically.

The best results for each factor were combined in one experiment, and the overall lactic acid released increased by 62%. Further work is necessary to find the best conditions, making use of statistical methods, such as factorial designs, and controlled reaction conditions in the reactor (pH control, for example) and improvements in mass transfer should be studied (the type and speed of agitation).

There are not many reports on the mechanism of PLA degradation by microorganisms or enzymes, except for the work by Kitadoro [30], which refers to the catalytic site, but not to the type of cleavage on the polymer chain. Enzymes such as the cutinases used in this work could break ester bonds and generate oligomers or monomers, and, in commercial packaging, the presence of additives could promote or hinder this action [31]. It is important to note that PLA breakdown does not depend directly on the activity of the enzyme, but there must be a difference in the interactions between the enzyme and substrate among the three cutinases and Proteinase K. What we know is that their action on cutin is different, as they yield different reaction products [18], but the interaction with other polyesters requires more research. As shown in Figure S1, the structures of the three cutinases are slightly different. Figure S1 shows the differences in superficial hydrophobicity and electrostatic potential at the reaction pH. Moreover, the recognition sites in ANCUT1 have the residues Leu189, Phe 92, Ile 191, and Leu 88. For ANCUT2, they are Leu 192, Val 194, Ala 95, and Leu 91, while for ANCUT3, they are Leu 195, Ala 99, Ile 197, and Leu 95, according to the models reported in UNIPROT. It must be noted that these residues are different to those described for the interaction between PLA and *Thermobifida fusca* cutinase, described by Kitadoro [30], which include several aromatic residues besides the catalytic His and Ser commonly found in esterases and cutinases. Therefore, the action mechanism of ANCUT1 on PLLA should be further investigated by performing a reaction kinetics study, and the products should be analyzed by ultra-high-performance liquid chromatography–electrospray ionization in high resolution to detect the reaction products. Among these, we might find lactide, lactoyllactic acid, dimers, and low-molecular-weight oligomers, as found by Mirsty [32]. These authors describe the biodegradation of PLA by a microbial community and consider the effect that the crystallinity of the substrate has in the degradation reaction.

They also describe random chain scissions, or what they call “backbiting” hydrolysis, which corresponds to different endolytic mechanisms. This can be compared to the phenomenon that Schubert et al. [33] report, where some PET hydrolases start their action on the polymer with endolytic cleavages and, when short oligomers appear, they combine endo- and exolytic mechanisms.

The results obtained indicate there is monomer release, but the total hydrolysis of the polymer is not achieved. According to a theoretical calculus that considers the molecular weight of the polymer used in this work, the lactic acid released corresponds to 37.69% of the total monomers of lactic acid present in the polymer. All the products must be identified to determine the final degree of hydrolysis. Moreover, longer incubation times could be explored to determine whether higher hydrolysis can be achieved. The preliminary results obtained with ANCUT3 indicate that this is not the case.

Physical damage to the polymer was observed through SEM. The magnitude of the damage seems to be correlated with the release of L lactic acid. Oligomer release and a decrease in molecular weight in the PLLA chains were not studied.

## 5. Conclusions

Three recombinant cutinases from *Aspergillus nidulans* were evaluated for PLA degradation in order to obtain the monomer, lactic acid. ANCUT1 released the higher concentration (41.84 mM) compared to the other two and to proteinase K. Some reaction parameters were evaluated and integrated in an experiment designed to improve reaction conditions. The results show an important increase in released lactic acid compared to proteinase K (483%). Weight loss was also improved, both in a chemically pure polymer and in a commercial package. ANCUT1 is not enantioselective and acts on both PDLA and PLLA. The SEM outcomes show different modes of action for each cutinase.

In summary, ANCUT1 seems to be a promising enzyme to achieve PLA degradation, which results in lactic acid recovery. Work is needed to optimize the reaction conditions, particularly at reactor levels. More studies need to be performed to understand the interaction between the enzyme and the substrate, as well as the precise identification of the products, to propose a degradation mechanism. Molecular tools may be used to improve its affinity towards the substrate and release higher amounts of the monomer. Other areas of research include the recovery of the lactic acid formed and its transformation into value-added products, to perform a circular economy process.

## Figures and Tables

**Table 1:** Protein concentration and esterase activity for recombinant cutinases expressed in *Pichia pastoris*.

Enzyme	Protein (mg/mL)	Volumetric Activity (U/mL)	Specific Activity (U/mg)
ANCUT1	0.180	1716.23	17,015.30
ANCUT2	0.152	6974.90	64,486.00
ANCUT3	0.168	180.92	3664.10

**Table 2:** Weight loss after 7 days of enzyme treatment.

Treatment	Weight Loss (%)
PLLA with no enzyme (blank)	0
PLLA-ANCUT 2	7.43 ± 0.53
PLLA-ANCUT3	6.93 ± 0.35
PLLA-ANCUT1	45.96 ± 1.13
PDLA-ANCUT 1	36.37 ± 1.35
Commercial package–ANCUT1	49.7 ± 1.50

## Data Availability

The ANCUT1 gene sequence is available at GenBank submission ID2808472.

## Supplementary Materials

The following supporting information can be downloaded at: https://www.mdpi.com/article/10.3390/polym16141994/s1, Figure S1: Representation of the hydrophobic surface of ANCUT1, ANCUT2, ANCUT3, and Proteinase K. References [34,35,36] are cited in the Supplementary Materials.
