# Supplementary material for: Poly(lactic acid) Degradation by Recombinant Cutinases from Aspergillus nidulans
Source: Polymers (Basel). 2024 Jul 12;16(14):1994. doi: 10.3390/polym16141994 (PMC11281152; doi:10.3390/polym16141994)
Supplement: Supplementary file 1 [file polymers-16-01994-s001.zip › polymers-3065115-supplementary.pdf]

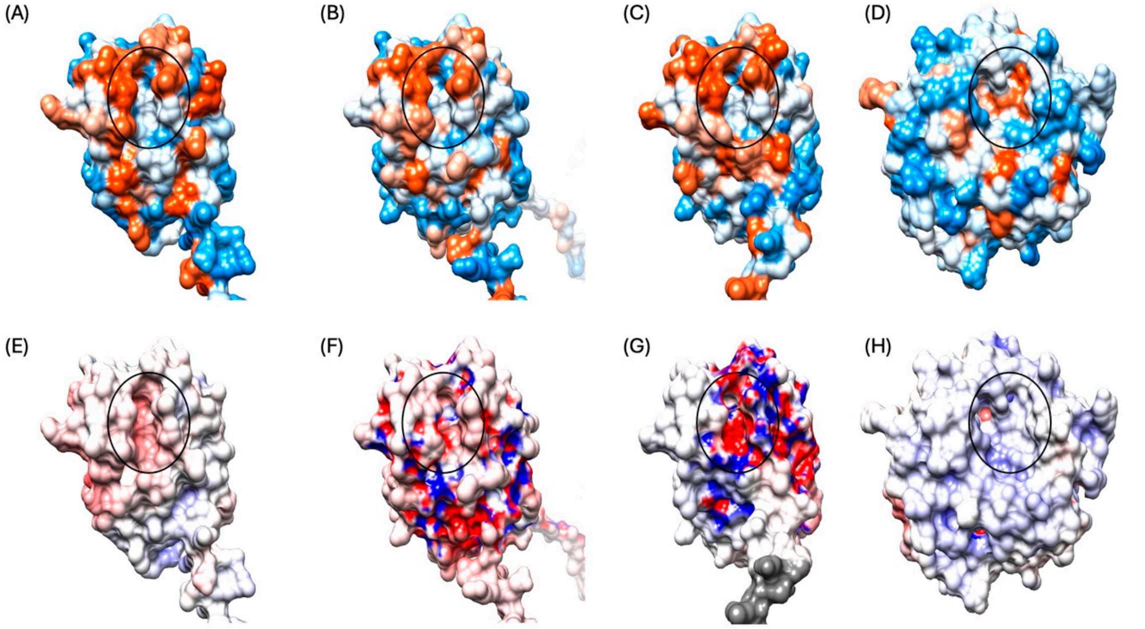


**Figure S1.** Representation of the hydrophobic surface of ANCUT1(UNIPROT ID: Q5B2C1) (A), ANCUT2 (UNIPROT ID: Q5AVY9) (B), ANCUT3 (UNIPROT ID: Q5AX00) (C), and Proteinase K (PDB ID: 6K2S) (D) (blue represents hydrophilic residues and red hydrophobic residues). The electrostatic potential was calculated using APBS web service (Jurrus et al., 2018) for ANCUT1 at pH 9 (F), ANCUT2 at pH 7 (G), ANCUT3 at pH 9 (H), and Proteinase K at pH 8 (I) (blue represents positive charge and red negative charge). The black circles show the active site. Visualized with Chimera (Pettersen et al., 2004). The cutinase models were taken from UNIPROT database and were predicted by AlphaFold (Jumper et al., 2021).

## References

1. Jurrus, E.; Engel, D.; Star, K.; Monson, K.; Brandi, J.; Felberg, L.E.; Brookes, D.H.; Wilson, L.; Chen, J.; et al. Improvements to the APBS biomolecular solvation software suite. *Protein Sci. A Publ. Protein Soc.* 2018, *27*, 112–128. https://doi.org/10.1002/PRO.3280
2. Pettersen, E.F.; Goddard, T.D.; Huang, C.C.; Couch, G.S.; Greenblatt, D.M.; Meng, E.C.; Ferrin, T.E. UCSF Chimera—A visualization system for exploratory research and analysis. *J. Comput. Chem.* **2004**, *25*, 1605–1612. https://doi.org/10.1002/JCC.20084
3. Jumper, J.; Evans, R.; Pritzel, A.; Green, T.; Figurnov, M.; Ronneberger, O.; Tunyasuvunakool, K.; Bates, R.; Žídek, A.; Potapenko, A.; et al. Highly accurate protein structure prediction with AlphaFold. *Nature* **2021**, *596*, 583–589. https://doi.org/10.1038/s41586-021-03819-2
